# A simple solution to deliver a second guidewire without a dual‐lumen microcatheter—The sidecar technique

**DOI:** 10.1002/ccr3.4787

**Published:** 2021-09-09

**Authors:** Gregor Leibundgut, Heinz‐Joachim Büttner

**Affiliations:** ^1^ Department of Cardiology Medizinische Universitätsklinik Kantonsspital Baselland Liestal Switzerland; ^2^ Klinik für Kardiologie und Angiologie II Universitäts‐Herzzentrum Freiburg‐Bad Krozingen Bad Krozingen Germany

**Keywords:** bifurcations, chronic total occlusions, complex PCI, dissections, dual‐lumen microcatheter, guidewire co‐transporter

## Abstract

The sidecar technique represents a simple and inexpensive solution to successfully deliver a second guidewire distally whenever a dual‐lumen microcatheter is not available.

## Graphical abstract

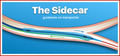

## 1 INTRODUCTION

Complex percutaneous coronary interventions include bifurcation lesions and tortuous anatomy that may need additional tools for successful procedures. Dual‐lumen microcatheters provide particular features and should be part of the toolbox of every modern catheterization laboratory. The sidecar technique represents a simple and inexpensive alternative solution.

After successful wire passage and extensive predilatation, multiple dissection planes may complicate the passage of a second guidewire for side branch wiring or additional support. Other scenarios include tight lesions, chronic total occlusions (CTO), tortuous anatomy, bifurcations, dissections, and preventing wire twist. Dual‐lumen microcatheters have been introduced and successfully used in these situations. However, not all catheterization laboratories have access to these devices due to various reasons (financial, legal, registration, operator's choice, etc.). For those situations where you need a guidewire co‐transporter to successfully deliver a second guidewire distally, we introduce the sidecar technique. Everyone can make its own sidecar co‐transporter from a regular balloon catheter.

## 2 CASE REPORT

An 80‐year‐old male patient with history of hypertension and dyslipidemia presented to our emergency department with new onset of chest pain and a non‐ST segment elevation myocardial infarction (NSTEMI) due to a subtotal occlusion of the distal right coronary artery (RCA). Echocardiography showed concentric left ventricular hypertrophy and an inferior wall motion abnormality with mildly impaired left ventricular function (LVEF 44%).

For the intervention of the RCA, a guidewire was passed through the severe stenosis of the distal RCA into the posterolateral branch (PLB). Extensive balloon dilatation of the vessel resulted in large dissection planes, which complicated the passage of a second guidewire to protect the posterior descending artery (PDA). Initial wiring of the PDA was considered unnecessary but became crucial after the dissection extended into the distal bifurcation of the PLB and PDA. A dual‐lumen microcatheter was not available at that time, and the operator was unable to deliver the second guidewire distally passing the dissection planes. He then came up with a guidewire co‐transporter made from a regular 2.0/20 SC balloon catheter that was used to predilate the lesion and was able to successfully place a second guidewire into the PDA.

**FIGURE 1:**
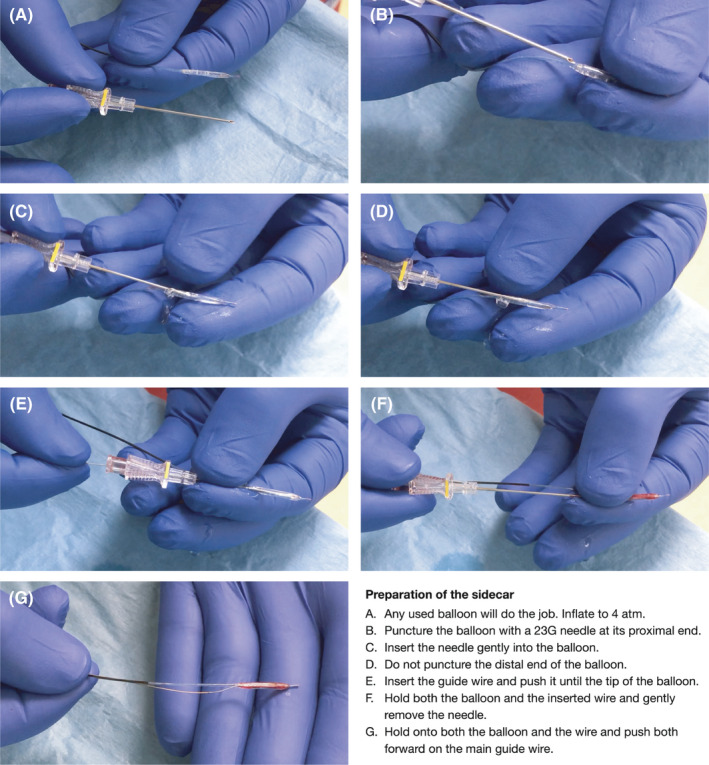
Preparation of the sidecar. (A) Any used balloon will do the job. Evacuate air from the balloon as indicated in the IFU and inflate with saline to 4 atm. (B) Puncture the inflated balloon with a 23G needle at its proximal end. (C) Insert the needle gently into the balloon. (D) Do not puncture the distal end of the balloon. (E) Insert the guidewire and push it forward until the tip of the balloon. (F) Hold both the balloon and the inserted wire and gently remove the needle. (G) Hold onto both the balloon and the wire and push both forward on the main guidewire. Never inflate again over the balloon hub to avoid air embolization

Preparation of the guidewire co‐transporter (sidecar) is illustrated step‐by‐step in Figure 1. Any regular balloon catheter can be used with longer balloons being more practical. Inflate the balloon to 4 atm and puncture it at its proximal end with a 23‐gauge needle (radial puncture needles are ideal). Insert the needle gently into the balloon without puncturing the distal end of the balloon. Insert the guidewire through the needle into the balloon and push it until the tip of the balloon. Squeeze all the air and contrast media out of the balloon by sliding your fingers from distal to proximal while holding the second guidewire together with the balloon shaft. Hold both the balloon and the inserted second guidewire between your right thumb and index finger and gently remove the needle with your left hand. Thread the balloon onto the main guidewire and push both the balloon and the second guidewire forward together.

**FIGURE 2:**
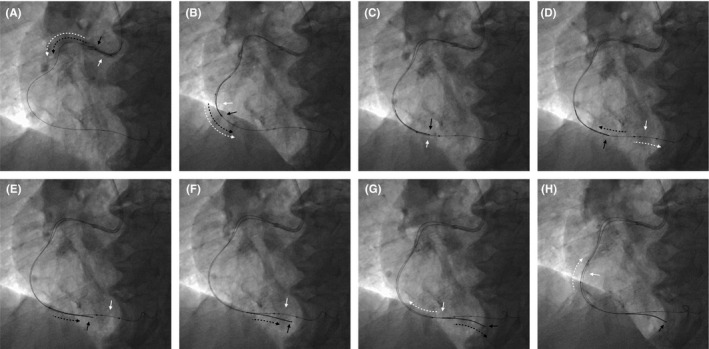
The sidecar technique. (A) Advance the balloon together with the second guidewire inside over the main guidewire. (B) Keep pushing both the balloon and the second guidewire together to the distality. (C) Push the balloon slightly past the side branch. (D) Advance the balloon while holding the second guidewire in place or slightly retract it a little (unparking). (E) Advance the second guidewire past the balloon. (F) Advance the second guidewire into the side branch. (G) Retract the balloon from the distality. (H) Completely remove the balloon from the coronary artery. White arrows indicate position and moving direction of the balloon. Black arrows indicate the tip of the second guidewire and its moving direction

The interventional procedure of the sidecar maneuver is demonstrated step‐by‐step in Figure 2. Advance the balloon through the lesion as far distal as possible. Retract the second guidewire from the balloon and advance it again outside parallel to the balloon. Then remove the balloon catheter from the coronary artery.

**FIGURE 3:**
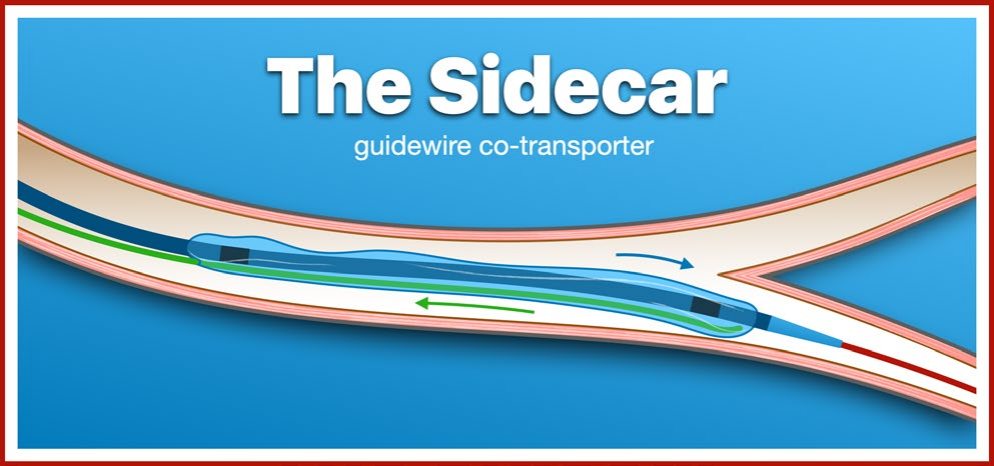
The sidecar

The cartoon (Figure 3) illustrates the removal of the second guidewire from the sidecar once the distality has been reached. Moving images of both the preparation of the guidewire co‐transporter (Video S1) and the entire sidecar technique (Video S2) are available in the online appendix.

As an alternative to the sidecar technique, one can also puncture the distal end of the balloon and let the guidewire exit the balloon at the distal end. This may be needed in certain situations such as reverse wiring or puncture of an occluded side branch (slipstream technique).^1^

## 3 DISCUSSION

We have performed five different cases successfully using this technique.

In the first three cases, the sidecar technique was used to facilitate the passage of a second guidewire through a previously balloon dilated and severely dissected proximal lesion that was difficult to wire. The technique not only enables successful wire passage but also reduces the risk of propagating dissections with the second guidewire.

In a fourth case, the sidecar significantly reduced wire twist in a very tortuous vessel and simplified bifurcation stenting.

In a fifth case, we adapted the balloon preparation with an additional distal puncture to let the second guidewire pass completely through the balloon to solve a retroflex take‐off of a side branch with reverse wiring.

Dual‐lumen microcatheters have become widely available and should be part of the toolbox in modern catheterization laboratories. However, various reasons (financial, legal, registration, operator's choice, etc.) may preclude the availability in certain situations. The sidecar technique is readily available in every catheterization laboratory and may present an alternative in these circumstances. Foremost, it comes at no additional cost, especially when a previously used balloon is recycled. An advantage over conventional dual‐lumen microcatheters is the missing over the wire (OTW) part which facilitates removal once the second guidewire was placed. The sidecar technique works with any balloon sizes as long as the operator is able to puncture the inflated balloon with a 23G needle. Although more complicated, we have been able to puncture and insert a guidewire into a 1 mm balloon. Smaller balloons may be favorable to cross tight lesions such as chronic total occlusions.

## 4 LIMITATIONS

The sidecar technique may be limited in very tight lesions and heavily calcified and long CTO lesions due to the less favorable crossing profile and pushing capabilities compared to a dual‐lumen microcatheter. In such lesions, prior passage of a regular microcatheter may be helpful to create a small enough channel for the balloon co‐transporter.

Stearability and directability of the second guidewire may be limited when compared to a dedicated dual‐lumen microcatheter.

Attachment of the co‐transporter's hub to the indeflator should be avoided at any point the balloon catheter is inside the coronary artery to prevent air embolization. Without actively injecting, there is no risk of air getting out into the vasculature due to the positive arterial pressure, even in occluded segments. The blood will always flow back into the catheter and push back any small amounts of air remaining in the catheter. Importantly, the balloon catheter should always be aspirated and filled with either saline or contrast media before being punctured.

Failure to retrieve the balloon in calcified lesions is a possible complication that is minimized due to the fact that the crossing profile does not change between advancing and retrieving the balloon. Even more, the crossing profile seems to be favorable without the second wire inside after successful distal deployment and subsequent removal of the second wire. The use of smaller balloon diameters is recommended in tight lesions and CTOs.

Losing the wire during distal transportation is possible. In this situation, either the second wire has already advanced distal enough and the co‐transporter can be removed, or the wire has to be removed followed by the co‐transporter, with the whole process to be performed again.

## 5 CONCLUSION

The sidecar technique represents a simple and inexpensive solution whenever a dual‐lumen microcatheter is not available.

## CONFLICT OF INTEREST

None declared.

## AUTHOR CONTRIBUTION

Both authors conceived the idea and wrote the manuscript.

## ETHICAL APPROVAL

This material is the authors' own original work, which has not been previously published elsewhere.

## Supporting information

Video S1

Video S2

## Data Availability

Data sharing not applicable to this article as no datasets were generated or analysed during the current study.
